# HAIC Combined with lenvatinib plus PD-1 versus lenvatinib Plus PD-1 in patients with high-risk advanced HCC: a real-world study

**DOI:** 10.1186/s12885-024-12233-6

**Published:** 2024-04-16

**Authors:** Xu Chang, Xinge Li, Peng Sun, Zhenfeng Li, Pengfei Sun, Shangkun Ning

**Affiliations:** 1 Department of Interventional Therapy II, Shandong Cancer Hospital and Institute, Shandong First Medical University, Shandong Academy of Medical Sciences, Jinan, Shandong China; 2 Department of Oncology, Central Hospital Affiliated to Shandong First Medical University, Shandong First Medical University, Shandong Academy of Medical Sciences, Jinan, Shandong China; 3 Department of Interventional Therapy I, Shandong Cancer Hospital and Institute, Shandong First Medical University, Shandong Academy of Medical Sciences, 250117 Jinan, Shandong China; 4 Department of Hepatological Surgery, Shandong Cancer Hospital and Institute, Shandong First Medical University, Shandong Academy of Medical Sciences, 250117 Jinan, Shandong China

**Keywords:** HCC, HAIC, Lenvatinib, PD-1, High-risk

## Abstract

**Background:**

The treatment of hepatocellular carcinoma (HCC) patients exhibiting high-risk characteristics (Vp4, and/or bile duct invasion, and/or tumor occupancy ≥ 50%) lacks standardized approaches and yields unfavorable results. This study endeavors to evaluate the safety, efficacy, and prognostic impacts of employing hepatic arterial infusion chemotherapy (HAIC), lenvatinib, and humanized programmed death receptor-1 (PD-1) in the treatment of high-risk HCC patients.

**Methods:**

In this retrospective analysis, HCC patients with high-risk features were treated with either lenvatinib combined with PD-1 (LEN-PD1) or a combination of HAIC, lenvatinib, and PD-1 (HAIC-LEN-PD1). The study assessed the antitumor efficacy by calculating overall survival (OS), progression-free survival (PFS), objective response rate (ORR), and disease control rate (DCR). Treatment-related adverse events (TRAEs) were analyzed to assess the safety profiles.

**Results:**

Between June 2019 and September 2022, a total of 61 patients were included in the LEN-PD1 group, while 103 patients were enrolled in the HAIC-LEN-PD1 group. The OS was 9.8 months in the LEN-PD1 group, whereas the HAIC-LEN-PD1 group exhibited a significantly longer median OS of 19.3 months (HR = 0.43, *p <* 0.001). Furthermore, PFS was notably extended in the HAIC-LEN-PD1 group compared to the LEN-PD1 group (9.6 months vs. 4.9 months, HR = 0.48, *p* < 0.001). Patients in the HAIC-LEN-PD1 group had a higher ORR and DCR according to the modified RECIST (76.7% vs. 23.0%, *p* < 0.001; 92.2% vs. 72.1%, *p* = 0.001). HAIC-LEN-HAIC group led to more adverse events than LEN-PD1 group, most of which were tolerable and controllable.

**Conclusion:**

Lenvatinib, HAIC and PD-1 showed safe and promising anti-tumor activity compared with lenvatinib alone for HCC with high-risk features.

**Supplementary Information:**

The online version contains supplementary material available at 10.1186/s12885-024-12233-6.

## Key points

• HAIC-LEN-PD1 combination treatment for HCC.

• HAIC-LEN-PD1 is a safe and effective treatment for high-risk patients with HCC.

• HAIC-LEN-PD1 is more effective than LEN-PD1 in prolonging patient survival.

**Supplementary Information:**

The online version contains supplementary material available at 10.1186/s12885-024-12233-6.

## Graphical abstract

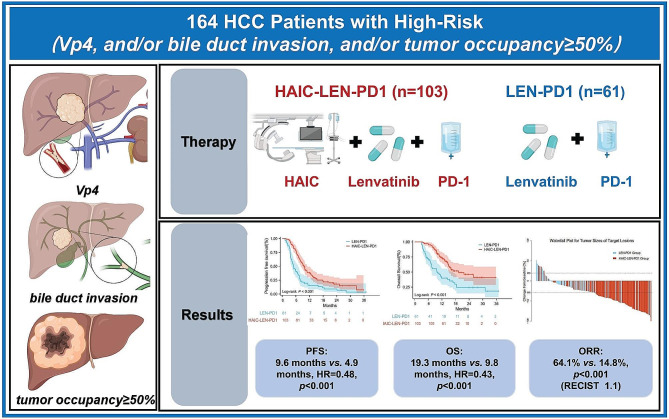

## Introduction

Hepatocellular carcinoma (HCC) is a prevalent malignancy, ranking as the sixth most frequently diagnosed cancer and the third leading cause of cancer-related mortality globally [1, 2]. The burden of HCC is particularly high in Asia, where approximately 72% of all HCC cases are reported, with China alone accounting for more than 50% [3]. Unfortunately, most patients are diagnosed at advanced stages when curative surgery is not feasible, resulting in a discouraging 5-year survival rate ranging from 10 to 18% [4, 5].

First-line systemic therapies for unresectable advanced HCC currently include tyrosine kinase inhibitors (TKIs), such as sorafenib (SHARP) [6] and lenvatinib (REFLECT) [7]. Recently, the Food and Drug Administration (FDA) approved a combination of atezolizumab and bevacizumab for patients with unresectable or metastatic HCC who had not undergone prior systemic therapy, based on the phase III IMbrave150 trial [8]. Subsequently, this is recommended as the preferred first-line approach by various guidelines owing to its remarkable efficacy. However, the latest updated findings from IMbrave150 have revealed limited benefits for high-risk patients, characterized by Vp4, bile duct invasion, and/or tumor occupancy ≥ 50% (TO ≥ 50%) of the liver, with a median overall survival (OS) of 7.6 months [9, 10]. Unfortunately, patients with major portal vein tumor thrombosis (PVTT) (Vp3 and Vp4) treated with sorafenib experienced a disappointingly short median survival of only 3.1–6.0 months [11, 12]. Notably, high-risk patients were not included in the REFLECT trial. Outcome data of high-risk patients are limited because of their extremely poor prognosis, resulting in often being excluded from previous trials. Consequently, there is an unmet need for effective interventions for high-risk patients.

Recently, a few combination immunotherapy approaches, such as lenvatinib plus pembrolizumab (KEYNOTE-524) [13] and camrelizumab plus apatinib (CARES-310) [14], have emerged as first-line treatment options for patients with HCC. Although the phase III LEAP-002 trial, which evaluated the combination of lenvatinib and pembrolizumab, did not achieve its intended primary endpoint it should be noted that this treatment regimen exhibited superior OS compared to lenvatinib alone (21.2 vs.19.0 months, hazards ratio [HR] = 0.84; 95% confidence interval [CI]: 0.708–0.997, *p* = 0.0227). These combination therapies have demonstrated promising anti-tumor activity and displayed acceptable safety profiles when used in treatment-naïve patients with unresectable HCC [15]. These findings emphasize the importance of further investigation into the synergy between TKIs and immune checkpoint inhibitors (ICIs).

Hepatic arterial infusion chemotherapy (HAIC), a therapeutic approach employed for primary and metastatic hepatic malignant tumors, enables the targeted delivery of a potent dosage of drugs directly to liver tumors [16–19]. This localized administration results in a significant local anti-tumor effect. Recent investigations have focused on the use of HAIC as a stand-alone treatment or in combination with sorafenib for advanced HCC, with notable findings being favorable outcomes in terms of both response rate and survival outcomes [16, 18, 20].

Considering the different anti-malignant mechanisms of TKIs, PD-1 inhibitors, and HAIC, combining these three modalities may show potential synergistic effects and promising preliminary efficacy in advanced HCC. This study aimed to retrospectively investigate the effectiveness and safety of combining lenvatinib with PD-1 inhibitors and HAIC as a first-line treatment for high-risk patients with advanced HCC.

## Materials and methods

### Patients

We retrospectively reviewed candidate patients with high-risk HCC who received either lenvatinib and PD-1s or the combined treatment HAIC plus lenvatinib and PD-1s at the Affiliated Cancer Hospital of Shandong First Medical University between June 2019 and September 2022 (Supplementary material 1a). All patients were diagnosed with HCC based on either noninvasive criteria or biopsies. The noninvasive diagnostic criteria were: liver cirrhosis, tumor diameter > 1 cm based on four-phase multidetector computed tomography (MDCT) or dynamic magnetic resonance imaging, and arterial hypervascularization with venous or delayed phase washout. The inclusion criteria were as follows: (1) age between 18 and 80 years; (2) liver function classification (Child-Pugh) of grade A or B; (3) Eastern Cooperative Oncology Group score 0–2; (4) presence of Vp4, and/or bile duct invasion and/or tumor occupancy ≥ 50% of the liver; (5)at least one measurable intrahepatic lesion according to Response Evaluation Criteria in Solid Tumors (RECIST) version 1.1; (6) no prior systemic options performed; and (7) and adequate organ function (absolute neutrophil count ≥ 1.5 × 10^9^/l, platelet count ≥ 60 × 10^9^/l, total bilirubin < 52 µmol/L, albumin ≥ 28 g/l, aspartate transaminase and alanine transaminase ≤ 5 × upper limit of the normal, creatinine clearance rate of ≤ 1.5 × upper limit of the normal, and left ventricular ejection ≥ 45%).The exclusion criteria were as follows: the presence of other malignant tumors, incomplete medical information, Child-Pugh C, and loss to follow-up. This single-center retrospective study was approved by the Ethics Committee of the Affiliated Cancer Hospital of Shandong First Medical University (SDTHEC 2,023,004,006). Written informed consent was obtained from all patients before the operation, and all procedures were in accordance with the 1955 Declaration of Helsinki.

### Treatment

Each patient received oral lenvatinib at 12 mg/day ( body weight > 60 kg) or 8 mg/day ( body weight < 60 kg). Patients received lenvatinib as described above 3–5 days before the initial HAIC to confirm tolerability. Dose reductions owing to lenvatinib-related toxicities (to 8, 4, or 4 mg every other day) were allowed. Sintilimab, camrelizumab, and tislelizumab were administered intravenously as ICIs at a dose of 200 mg 0–1 day after HAIC. HAIC was performed every 3 weeks, and a catheter/microcatheter was placed in the main feeding hepatic artery. The following regimen was administered via the hepatic artery: oxaliplatin 135 mg/m^2^ from hour 0 to 2 on day 1; leucovorin 400 mg/m^2^ from hour 2 to 3 on day 1; 5-fluorouracil 400 mg/m^2^ bolus at hour 3; and 2400 mg/m^2^ over 46 h on days 1 and 2 (Supplementary material 1a). After completion of HAIC, the catheter and sheath were removed. Repetitive catheterization was performed during the subsequent HAIC cycles. HAIC was discontinued after 6 cycles, and the patients were treated with lenvatinib and PD-1 maintenance. Second-line systemic therapy or other palliative therapies should be adopted after first-line combination therapy failure. Second-line agents should be considered based on clinical judgment, toxicity profiles, and drug availability. Therapeutic decisions were made through multidisciplinary discussions among medical oncologists, surgeons, and radiologists.

### Follow-up

The primary endpoint was OS, defined as the time from the commencement of lenvatinib treatment to death from any cause. The secondary endpoint, progression-free survival (PFS), was defined as the time from the commencement of lenvatinib treatment to progression according to the modified RECIST (mRECIST) criteria or death from any cause, whichever occurred first. The objective response rate (ORR) was defined as the proportion of patients with a complete or partial response from the first radiological confirmation of that rate, and the disease control rate (DCR) was defined as the proportion of patients with ORR plus stable disease. DCR and ORR were evaluated according to the RECIST version 1.1 and mRECIST after 2 cycles of the combination therapy. Adverse events were assessed according to the National Cancer Institute Common Terminology Criteria for Adverse Events, version 5.0. Telephone follow-up and outpatient interview continued and ended on July 1, 2023. For patients without contact during follow-up, OS was calculated as the time from diagnosis to the last follow-up.

### Statistical analysis

The R Programming Language and SPSS (version 26.0) were used for all statistical analyses. Both OS and PFS were evaluated using the Kaplan–Meier method and log-rank test, with *p* < 0.05 defined as statistically significant. Cox proportional risk regression models were used for univariate and multivariate analyses. Univariate analysis was performed, and variables with *p* < 0.1 were selected for multivariate analysis, with *p* < 0.05 considered statistically significant.

## Results

### Patient characteristics and treatment

A total of 164 patients with high-risk HCC were included in this study. Among these, 103 received HAIC-LEN-PD1 treatment and 61 received LEN-PD1 treatment. Generally, both groups had balanced baseline characteristics. Patient characteristics are listed in Table 1. The HAIC-LEN-PD1 group comprised 12 females and 91 males with a median age of 52 years. The LEN-PD1 group comprised 4 females and 57 males with a mean age of 56 years. The median tumor size was 12.2 cm in the HAIC-LEN-PD1 group and 13.5 cm in the LEN-PD1 group. Most patients had Child-Pugh A disease (HAIC-LEN-PD1, *n* = 84; LEN-PD1, *n* = 50) and underlying chronic liver disease caused by hepatitis B virus infection (HAIC-LEN-PD1, *n* = 93; LEN-PD1, *n* = 58) who all received antiviral therapy. Extrahepatic spread was observed in 32 and 20 patients from the HAIC-LEN-PD1 and LEN-PD1 groups, respectively. In the LEN-PD1 group, 18, 31, and 12 patients had TO ≥ 50%, Vp4, and both TO ≥ 50% and Vp4, respectively. In the HAIC-LEN-PD1 group, 33, 54, and 16 patients had TO ≥ 50%, Vp4, and both TO ≥ 50% and Vp4, respectively. In addition, no patients with bile duct invasion was observed in this study.

**Table 1:** Baseline characteristics of the two group patients

Characteristics	HAIC-LEN-PD1 (*n* = 103)	LEN-PD1 (*n* = 61)	*P*
Gender Male Female	91 (88.3%) 12 (11.7%)	57 (93.4%) 4 (6.6%)	0.288
Age, years (median, SD)	52.0, 8.82	56.0, 7.88	0.115
ECOG score 0–1 2	96 (93.2%) 7 (6.8%)	58 (95.1%) 3 (4.9%)	0.627
BCLC B C	4 (3.8%) 99 (96.2%)	2 (3.3%) 59 (96.7%)	0.842
Child-Pugh A B	84 (81.6%) 19 (18.4%)	50 (82.0%) 11 (18.0%)	0.947
Viral status HBV others	93 (90.3%) 10 (9.7%)	58 (95.1%) 3 (4.9%)	0.272
Tumor size, cm (Mean) ≥ 10 cm < 10 cm	71 (68.9%) 32 (31.1%)	45 (73.8%) 16 (26.2%)	0.510
Portal vein invasion Absent VP1-2 VP3 VP4	9 (8.7%) 5 (4.9%) 19 (18.4%) 70 (68.0%)	4 (6.6%) 5 (8.2%) 9 (14.8%) 43 (70.4%)	0.882
Venous invasion Absent Present	76 (73.8%) 27 (26.2%)	50 (82%) 11 (18%)	0.230
Extrahepatic spread Absent Present	71 (68.9%) 32 (31.1%)	41 (67.2%) 20 (32.8%)	0.819
Tumor number Single Multiple	39 (37.9%) 64 (62.1%)	25 (41%) 36 (59%)	0.692
AFP, ng/ml(Mean) ≤ 400 > 400	33 (32.0%) 70 (68.0%)	26 (42.6%) 35 (57.4%)	0.172
High-risk type VP4 TO 50% Both	54 (52.4%) 33 (32.1%) 16 (15.5%)	31 (50.8%) 18 (29.5%) 12 (19.7%)	0.802
PD-1 inhibition agent Camrelizumab Tislelizumab Sintilimab	62 (60.2%) 26 (25.2%) 15 (14.6%)	32 (52.5%) 20 (32.8%) 9 (14.7%)	0.694
ALBI grade I II III	54 (52.4%) 47 (45.7%) 2 (1.9%)	25 (41.0%) 33 (54.1%) 3 (4.9%)	0.257
Laboratory tests TBil, µmol/L (Mean, SD) Albumin, g/L (Mean, SD) Platelet, ×109/L (Mean, SD) WBC, ×109/L (Mean, SD) PT, seconds (Mean, SD) Creatinine, µmol/L (Mean, SD) ALT, IU/L (Mean, SD) AST, IU/L (Mean, SD)	21.4, 12.3 38.4, 4.2 180.6, 78.6 6.0, 1.8 12.1, 1.1 65.5, 12.4 89.4, 55.9 58.1, 39.9	23.4, 14.4 39.3, 6.6 192.9, 86.6 6.4, 2.8 12.1, 1.1 75.0, 85.7 77.7, 38.6 49.9, 28.4	0.473 0.440 0.479 0.469 0.991 0.340 0.713 0.603

Abbreviations:

APF, α-fetoprotein; ALBI, albumin-bilirubin; ECOG, Eastern Cooperative Oncology Group; HBV, hepatitis B virus; HCV, hepatitis C virus; PCT, procalcitonin; IQR, interquartile range; ALT, alanine aminotransferase; AST, aspartate aminotransferase; TBil, total bilirubin; PT, prothrombin time; WBC, white blood cells

All 103 patients in the HAIC-LEN-PD-1 group received triple combination therapy with lenvatinib, PD1s, and HAIC. Among them, 62, 26, and 15 were treated with camrelizumab, tislelizumab, and sintilimab, respectively. In the LEN-PD1 group, 32, 20, and 9, were treated with camrelizumab, tislelizumab, and sintilimab, respectively.

### Efficacy

**Fig. 1:**
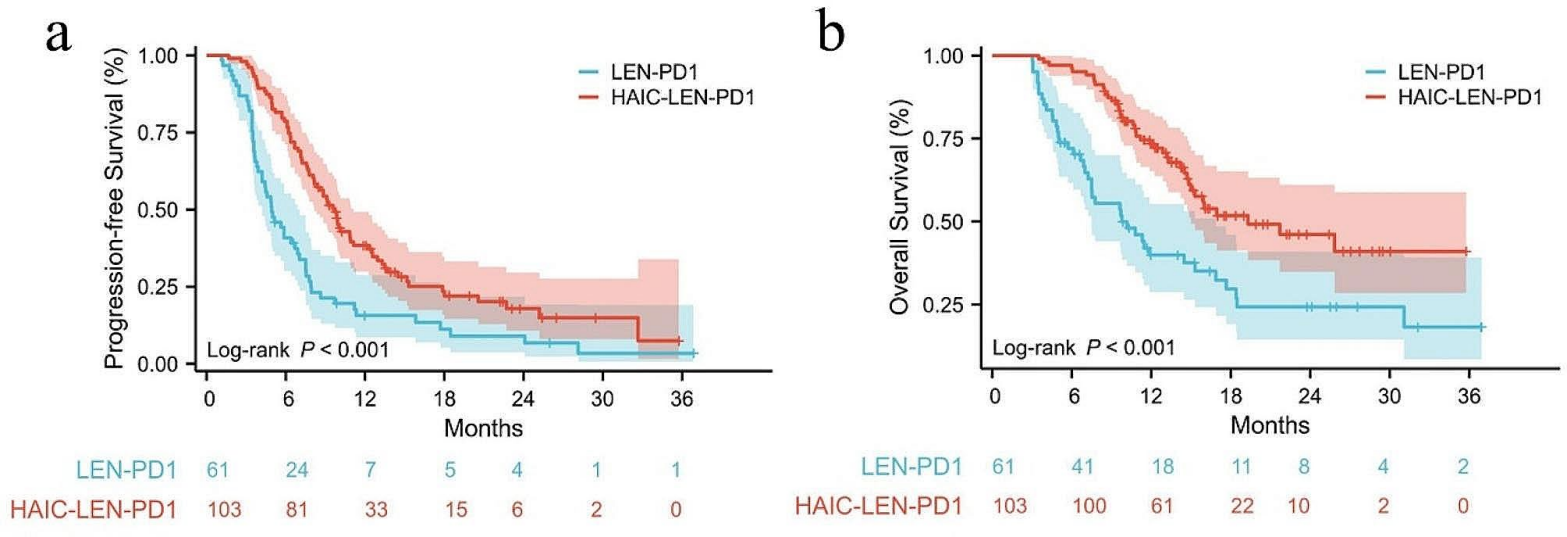
The progression-free survival (PFS) and overall survival (OS) of the two groups. Kaplan–Meier curves of PFS (a) and OS (b) for patients in the LEN-PD1 and HAIC-LEN-PD1 groups

The median follow-up time was 16.3 months in the HAIC-LEN-PD1 group and 24.1 months in the LEN-PD1 group. A total of 78 (75.7%) patients in the HAIC-LEN-PD1 group and 55 (90.2%) patients in the LEN-PD1 group experienced the disease progression or death. The median PFS time was significantly longer in the HAIC-LEN-PD1 group (9.6 months, 95%CI: 8.5–10.8) than the LEN-PD1 group (4.9 months, 95%CI: 3.6–6.1, HR = 0.48, *p <* 0.001) (Fig. 1a). A total of 83 deaths were observed (HAIC-LEN-PD1 *n* = 42; LEN-PD1 *n* = 41), and the median OS of the HAIC-LEN-PD1 and LEN-PD1 groups were 19.3 (95%CI: 11.0–27.5) and 9.8 (95%CI: 5.7–13.8) months, respectively (HR = 0.43, *p <* 0.001) (Fig. 1b).

**Fig. 2:**
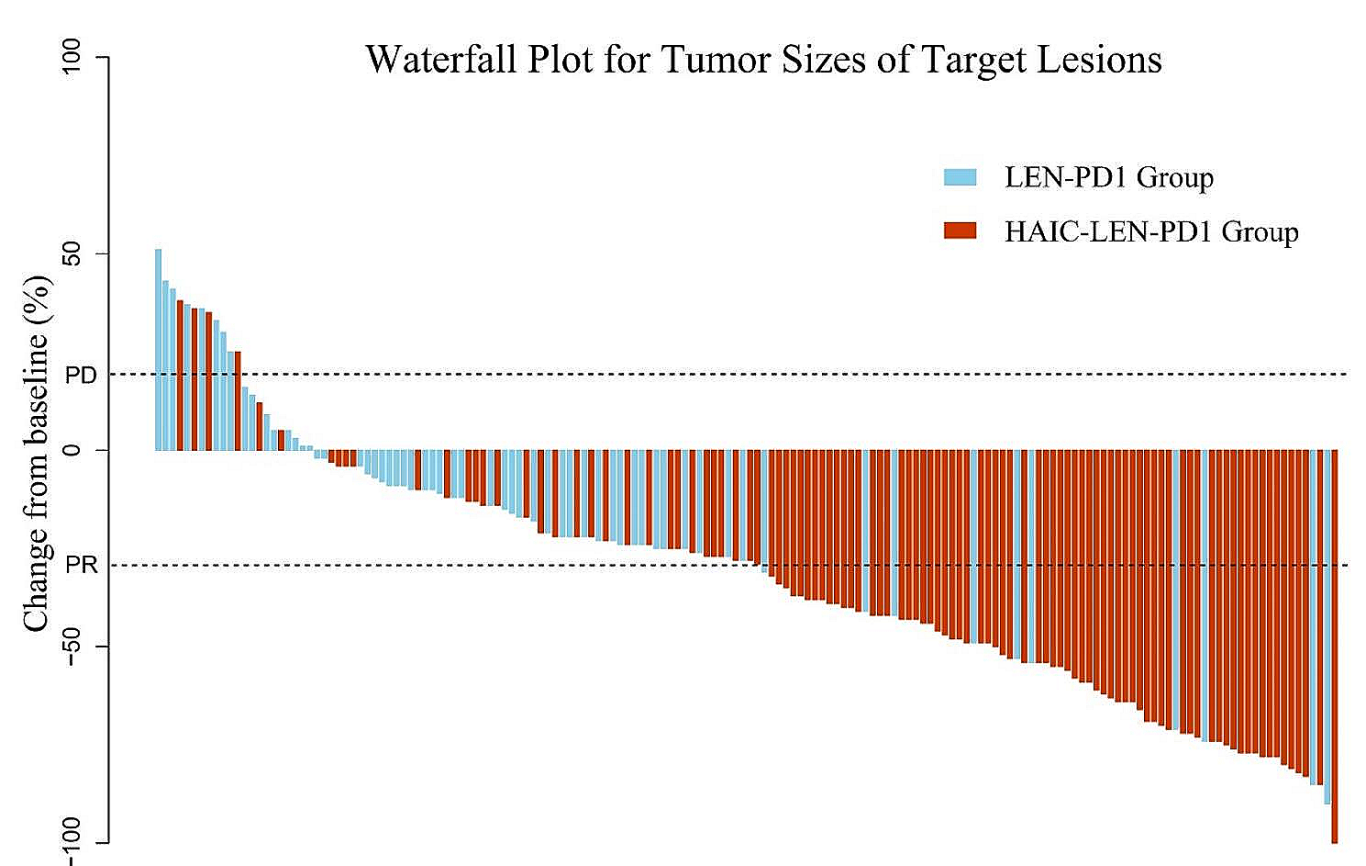
Waterfall plot for tumor size changes of intra-hepatic target lesions. Abbreviations: PD, progressive disease; PR, partial response

Tumor responses are listed in Table 2. Based on the RECIST1.1 criteria, the DCR and ORR were significantly higher in the HAIC-LEN-PD1 group than the LEN-PD1 group (92.2% vs. 72.1%, *p* = 0.001; 64.1% vs.14.8%, *p* < 0.001, respectively). Based on the mRECIST criteria, the DCR and ORR were also significantly higher in the HAIC-LEN-PD1 group than the LEN-PD1 group (92.2% vs.72.1%, *p* = 0.001 and 76.7% vs. 23.0%, *p* < 0.001, respectively). The intrahepatic tumor responses are presented in Table 2. The HAIC-LEN-PD1 group showed a significantly higher intrahepatic ORR than the LEN-PD1 group according to the mRECIST (80.6% vs. 24.6%, *p* < 0.001). The best response for intrahepatic target lesions according to the RECIST1.1 criteria is shown in the waterfall plot in Fig. 2. Additionally, 20 patients (HAIC-LEN-PD1, *n* = 18; LEN-PD1, *n* = 2) achieved a complete response to intrahepatic lesions based on the mRECIST.

**Table 2:** Summary of best response

	RECIST 1.1				mRECIST		
	HAIC-LEN-PD1	LEN-PD1	*P*-value		HAIC-LEN-PD1	LEN-PD1	*P*-value
**Overall Reponse**	*n* = 103	*n* = 61			*n* = 103	*n* = 61	
CR	1 (0.9%)	1 (1.6%)			16 (15.5%)	2 (3.3%)	
PR	65 (63.1%)	8 (13.1%)			63 (61.2%)	12 (19.7%)	
SD	29 (28.2%)	35 (57.4%)			16 (15.5%)	30 (49.1%)	
PD	8 (7.8%)	17 (27.9%)			8 (7.8%)	17 (27.9%)	
ORR	66 (64.1%)	9 (14.8%)	< 0.001		79 (76.7%)	14 (23.0%)	< 0.001
DCR	95 (92.2%)	44 (72.1%)	0.001		95 (92.2%)	44 (72.1%)	0.001
**Intrahepatic Response**	*n* = 103	*n* = 61			*n* = 103	*n* = 61	
CR	1 (0.9%)	1 (1.6%)			18 (17.5%)	2 (3.3%)	
PR	67 (65.1%)	9 (14.8%)			65 (63.1%)	13 (21.3%)	
SD	31 (30.1%)	36 (59.0%)			16 (15.5%)	31 (50.8%)	
PD	4 (3.9%)	15 (24.6%)			4 (3.9%)	15 (24.6%)	
ORR	68 (66.0%)	10 (16.4%)	< 0.001		83 (80.6%)	15 (24.6%)	< 0.001
DCR	99 (96.1%)	46 (75.4%)	< 0.001		99 (96.1%)	46 (75.4%)	< 0.001

CR, Complete response; PR, Partial response; SD, Stable disease; PD, Progressive disease; ORR, Objective responserate; DCR, Disease control rate

**Fig. 3:**
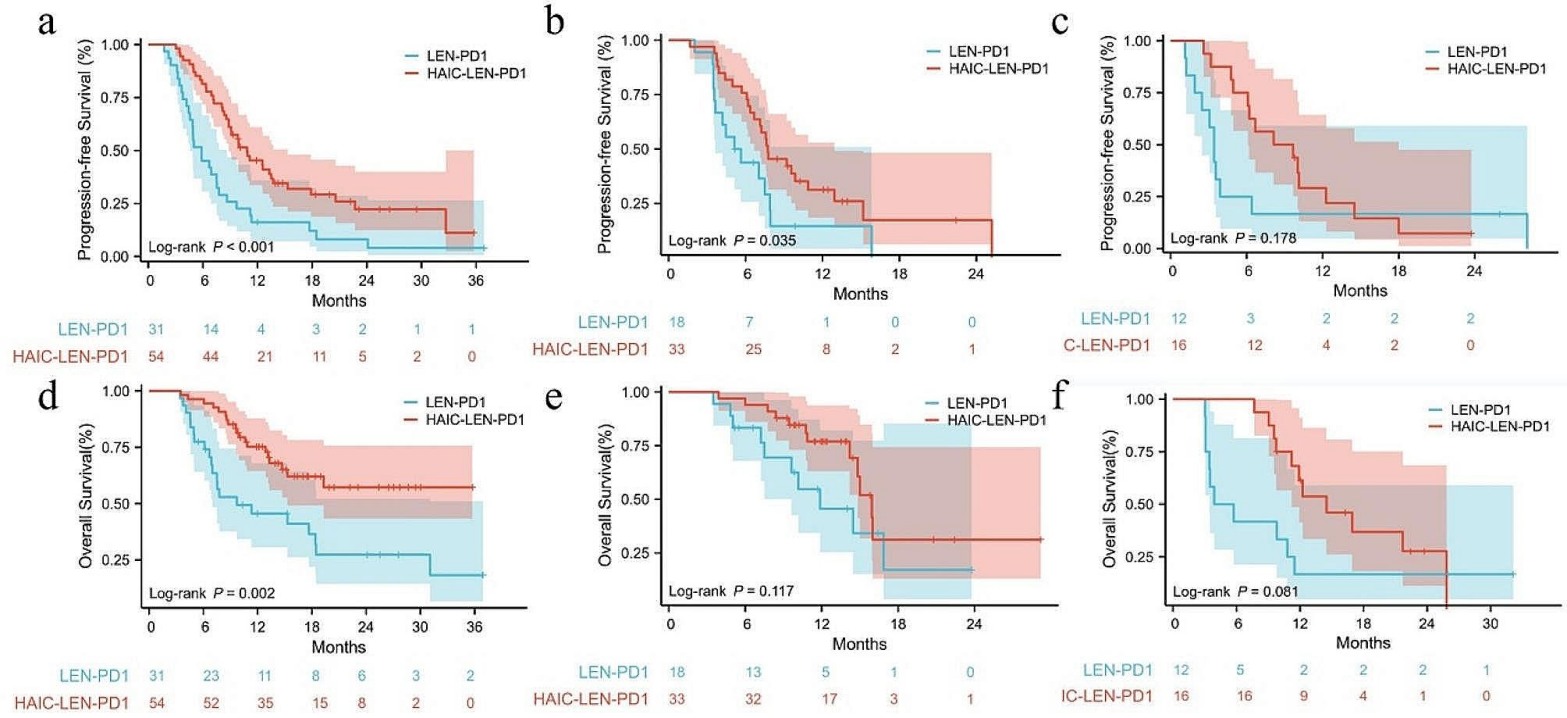
Survival outcomes of patients with different high-risk factors. (**a**), (**b**) and (**c**) were the PFS of patients with Vp4, TO ≥ 50% and both respectively. (**d**), (**e**) and (**f**) were the OS of patients with Vp4, TO ≥ 50% and both respectively

Additionally, we investigated whether there were differences in the efficacy of different high-risk features. The HAIC-LEN-PD1 group had superior OS (not estimable vs. 9.7 months, *p* = 0.002) and PFS (10.8 months vs. 5.8 months, *p* < 0.001) in patients with Vp4 than the LEN-PD1 group. For patients with TO ≥ 50%, the PFS was longer in the HAIC-LEN-PD1 group compared to the LEN-PD1 group (7.7 months vs. 5.1 months, *p* = 0.035), but there was no significant difference in OS (15.9 months vs. 11.8 months, *p* = 0.117). No significant difference was observed in PFS (8.1 months vs. 3.4 months, *p* = 0.178) and OS (14.5 months vs. 3.9 months, *p* = 0.081) between patients with both TO ≥ 50% and Vp4 although the HAIC-LEN-PD1 group had a much better PFS and OS. This maybe due to the small sample size of patients with these features (Fig. 3).

**Fig. 4:**
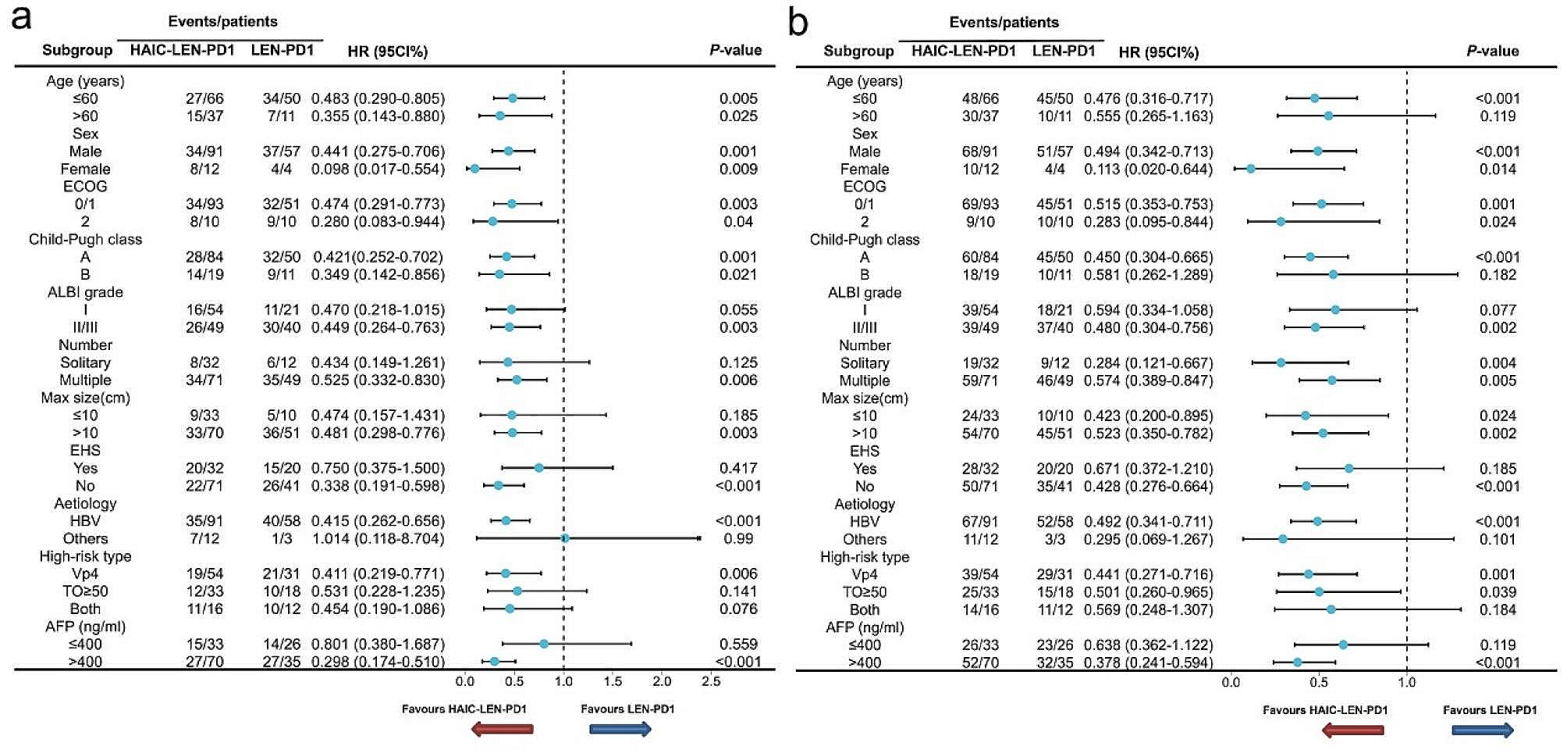
Forest plots of (**a**) overall survival and (**b**) progression-free survival in different patient subgroups. HR, hazard ratio; CI, confidence interval; ECOG, Eastern Cooperative Oncology Group; ALBI, grade Albumin-Bilirubin grade; AFP, alpha-fetoprotein; Vp, Japan’s portal vein invasion classification; EHS, extra-hepatic spread

We also compared the survival outcomes between patients in the HAIC-LEN-PD1 and LEN-PD1 group who received different types of PD-1s. There were no significant differences among the three types of PD1s, either in terms of median OS or PFS in HAIC-LEN-PD1 and LEN-PD1 group (Supplementary material 1b). Moreover, subgroup analysis showed that HAIC-LEN-PD1 was associated with better median OS and PFS than LEN-PD1 across most patient subgroups (Fig. 4).

The results of the univariate and multivariate analyses of PFS and OS are shown in Supplementary material 1c. Multivariate analysis showed that the independent risk factors for PFS were associated with HAIC or extrahepatic metastasis. Furthermore, the independent risk factors for OS were combined with HAIC, sex, and Child-Pugh Class.

In this study, 103 patients were treated with 396 HAIC cycles (median, 4 cycles). Dose adjustment of lenvatinib was observed in 28 patients in the HAIC-LEN-PD1 group and 12 patients in the LEN-PD1 group; however, none of the patients discontinued the target drugs. At the cutoff date, 78 and 55 patients developed disease progression in the HACI-LEN-PD1 and LEN-PD1 groups, respectively. Seventeen and 8 patients in the HACI-LEN-PD1 and LEN-PD1 groups, respectively, did not receive second-linetreatment because of refusal or poor liver function. The details of further treatments after disease progression are shown in Supplementary material 1d.

### Safety

No treatment-related deaths occurred (Table 3). The grade 3–4 adverse events (AEs) more common in the HAIC-LEN-PD1 group included alanine aminotransferase (ALT) elevation (20.4% vs. 8.2%, *p =* 0.039), neutropenia (11.7% vs.1.6%, *p =* 0.014), thrombocytopenia (16.5% vs.3.3%, *p =* 0.011), vomiting (9.7% vs.1.6%, *p =* 0.046), and hyperbilirubinemia (17.5% vs. 4.9%, *p =* 0.020). Any grade, including neutropenia, thrombocytopenia, ALT elevation, aspartateelevation, vomiting, and abdominal pain, was more frequent in the HAIC-LEN-PD1 than the LEN-PD1 group. For patients in the HAIC-LEN-PD1 group, the increases in serum aminotransferases and total bilirubin were most significant on the second day after the completion of HAIC, and most patients can recover to normal levels within one week. To better illustrate the effect of triple therapy on liver function, we have drawn a Sankey diagram of the dynamic changes in Child-Pugh classification for the two groups. As shown in Supplementary material 1e, the liver function in the HAIC-LEN-PD1 group deteriorates significantly after treatment. However, with proper hepatoprotective treatment, the majority of patients can recover their liver function to the pre-treatment level.

**Table 3:** Treatment related adverse events

Adverse event	HAIC-LEN-PD1			LEN-PD1			*P*-value	
	Any grade	Grade 3–4		Any grade	Grade 3–4		Any grade	Grade 3–4
Neutropenia	45 (43.7%)	12 (11.7%)		6 (9.8%)	1 (1.6%)		< 0.001	0.014
Anaemia	13 (12.6%)	2 (1.9%)		5 (8.2%)	0 (0.0%)		0.381	0.530
Thrombocytopenia	46 (44.7%)	17 (16.5%)		8 (13.1%)	2 (3.3%)		< 0.001	0.011
Fatigue	43 (41.7%)	3 (2.9%)		21 (34.4%)	2 (3.3%)		0.353	0.895
Hypertension	62 (60.2%)	9 (8.7%)		32 (52.5%)	5 (8.2%)		0.333	0.875
Weight loss	41(39.8%)	3 (2.9%)		21 (34.4%)	2 (3.3%)		0.492	0.895
Hypothyroidism	36 (34.9%)	3 (2.9%)		18 (29.5%)	2 (3.3%)		0.473	0.895
Rash	18 (17.5%)	5 (4.9%)		10 (16.4%)	2 (3.3%)		0.859	0.629
Vomiting	43 (41.7%)	10 (9.7%)		8 (13.1%)	1 (1.6%)		< 0.001	0.046
Diarrhea	15 (14.6%)	2 (1.9%)		6 (9.8%)	1 (1.6%)		0.472	0.889
Abdominal pain	62 (60.2%)	10 (9.7%)		12 (19.7%)	2 (3.3%)		< 0.001	0.213
Proteinuria	21 (20.4%)	5 (4.9%)		9 (14.8%)	2 (3.3%)		0.367	0.629
Elevated ALT	62 (60.2%)	21(20.4%)		11 (18.0%)	5 (8.2%)		< 0.001	0.039
Elevated AST	65 (63.1%)	21 (20.4%)		12 (19.7%)	6 (9.8%)		< 0.001	0.078
Hyperbilirubinacemia	45 (43.7%)	18 (17.5%)		15 (24.6%)	3 (4.9%)		0.087	0.020
Hyboalbuminaemia	42 (40.8%)	15 (14.6%)		23 (37.7%)	5 (6.6%)		0.697	0.229
Decreased appetite	38 (36.9%)	10 (2.9%)		20 (32.8%)	3 (4.9%)		0.595	0.895
Elevated creatinine	18 (17.5%)	4 (3.9%)		6 (9.8%)	1 (1.6%)		0.181	0.419
Immune-related hepatitis	5 (4.9%)	2 (1.9%)		2 (3.3%)	1 (1.6%)		0.859	0.889
Immune-related pneumonitis	3 (2.9%)	2 (1.9%)		2 (3.3%)	0 (0.0%)		0.629	0.530
Immune-related dermatitis Immune-related myocarditis	8 (7.8%) 1 (0.9%)	2 (1.9%) 0 (0.9%)		2 (3.3%) 0 (0.0%)	0 (0.0%) 0 (0.0%)		0.246 0.440	0.530 NA

Abbreviations: ALT, alanine aminotransferase; AST, aspartate; NA, notapplicable

Listed are adverse events, as defined by the National Cancer Institute Common Terminology Criteria (version 5.0)

The most common potentially immune-related TRAE was grade 1–2 hypothyroidism (34.9%). Moreover, 2 patients developed grade 3 immune-related dermatitis and 2 developed grade 3 immune-relatedhepatitis and pneumonitis. After treatment with corticosteroids and the suspending of PD1s, patients with immune-related dermatitis immediately returned to normal, and patients with immune-related hepatitis and pneumonitis recovered after 1 month. 6, 2 and 5 patients suspend of camrelizumab, sintilimab and tislelizumab due to 3–4 grade immune-related AEs. In addition, treatment related adverse events of different immune checkpoint inhibitors are shown in Supplementary material 1 f.

Additionally, the high incidence of abdominal pain may be associated with oxaliplatin infusion during the HAIC procedure; the specific abdomen could be acute and severe but was quickly relieved byimmediate infusion of lidocaine via a microcatheter.

Furthermore, we found that the spleen volume increased significantly more often in the HAIC-LEN-PD1 group during treatment than at baseline. Among them, 19 underwent partial splenic embolization.

## Discussion

Owing to the insidious onset of HCC, most patients are diagnosed at advanced stages, wherein a high tumor burden and PVTT often manifest as prevalent features. In China, patients with high-risk HCC tumor thrombi in the main portal vein trunk or those with a significant tumor burden, particularly those with tumors occupying more than 50% of the liver, are commonly encountered [3]. Unfortunately, these patients have a remarkably unfavorable prognosis [21, 22].

According to current clinical guidelines systemic therapy, such as lenvatinib or atezolizumab plus bevacizumab, is recommended as the first-line treatment for HCC. However, it is worth noting that the REFLECT, KEYNOTE-524, and LEAP-002 trials did not include patients with high-risk HCC, thus the safety and efficacy of lenvatinib in these patients are still uncertain [7, 13]. Additionally, the IMbrave150 study demonstrated that the combination of atezolizumab and bevacizumab provides limited benefit to high-risk patients, with a median OS of 7.6 months [10]. Consequently, available therapeutic options for patients with HCC with high-risk profiles are currently limited.

Recently, Japanese researchers discovered that lenvatinib has the potential to confer advantageous outcomes in high-risk patients. Estimated mPFS and mOS were 132 days and 229 days, and 101 days and 201 days in patients with TO ≥ 50% and Vp4, respectively [23]. Another study showed that lenvatinib plus PD-1can contribute to notable improvements in survival outcomes among patients with Vp4 and TO ≥ 50%, with the median OS was 11.39 and 6.1 months, respectively [24]. These studies demonstrate that lenvatinib is safe, effective, and could be a potential therapeutic approach for high-risk patients with HCC. In 2021, the phase III trial FOHAIC-1 demonstrated the heightened ORR and better survival outcomes of HAIC compared to sorafenib. The subgroup analysis unveiled that HAIC surpassed sorafenib even further in terms of OS and PFS in high-risk patients (10.8 vs. 5.7 months, 7.7 vs. 2.9 months respectively) [20]. Additionally, another phase II trial exploring the efficacy and safety of lenvatinib, toripalimab, and FOLFOX-HAIC as first-line treatments for advanced high-risk HCC showed promising results. The mPFS and mOS was 10.4 months and 17.4 months, respectively, and the ORR was as high as 66.7% [25]. These findings suggest that HAIC combined with systemic treatment may has potential synergistic efficacy and may be an alternative therapeutic option for high-risk patients with HCC; however, further studies are required to verify these observations. Hence, we conducted a retrospective study to assess the efficacy and safety of HAIC-LEN-PD1 versus LEN-PD1 as first-line treatment for high-risk patients with HCC.

Our previous single-arm study demonstrated the safety and efficacy of HAIC-LEN-PD1 therapy in high-risk HCC patients. The mPFS was 9.8 months and mOS were 19.3 months. According to the mRECIST, the ORR and DCR were 78.3% and 92.8%, respectively [26]. In this study, patients who received HAIC-LEN-PD1 achieved significantly better PFS (9.6 vs. 4.9 months) and OS (19.3 vs. 9.8 months) than patients who received lenvatinib and PD-1s. Previous studies have implied that the intrahepatic tumor burden affects survival outcomes in patients with advanced HCC, indicating that debulking of the liver tumor increases patient survival [27, 28]. Additionally, for patients with advanced HCC, it is crucial to reduce tumor burden and preserve liver function, especially in patients with a high tumor burden. Notably, the HAIC-LEN-PD1 group showed an ORR more than three times higher than that of the LEN-PD1 group (76.7% vs. 23.0%), which would be expected to improve liver function and allow the combined treatment to be continued for a longer time, which could potentially be attributed to the increased PFS and OS.

In our study, we chose HAIC as the locoregional treatment instead of TACE combined with lenvatinib plus PD-1s to rapidly reduce tumor burden. The reasons are as follows: first, in our study the median tumor size was 12.2 cm in the HAIC-LEN-PD1 group, while it has been demonstrated that the rate of complete tumor response was significantly lower in large (> 5 cm) HCCs than small HCCs (25% vs. 64%) [29]. Additionally, a large number of embolization particles are required to embolize large HCCs, which can increase the risk of deterioration of the hepatic functional reserve, post-embolization syndrome, and non-target embolization [30, 31]. A recent randomized phase III trial demonstrated the superior efficacy and safety of HAIC to TACE in patients with large unresectable HCC (OS: 23.1 vs.16.1 months). Second, most patients in our study had Vp4, which often leads to portal hypertension, a risk of esophagogastric varix rupture, and poor prognosis due to liver dysfunction. Unfortunately, traditional viewpoint holds that TACE is a relatively contraindication in patients with HCC complicated by Vp4, as it may disrupt the hepatic artery blood supply and lead to ischemia-related post-TACE liver failure. Therefore, almost no effective local treatment could be applied to patients with Vp4 currently [32]. Recently, several reports have shown that HAIC is an effective treatment for HCC with PVTT, even in patients with Vp4. Based on the aforementioned research, we deemed HAIC a more suitable local treatment option than TACE for patients with high-risk HCC.

Although patients treated with HAIC-LEN-PD1 had significantly elevated frequencies of grade 3–4 AEs, which may be due to the direct cytotoxicity to hepatocytes and hematopoietic cells induced by HAIC, these TRAEs were expected to be manageable by symptomatic treatment. Hypothyroidism, the most common immune-related adverse event, occurred in 34.9% of patients. All immune-related AEs disappeared after the participants stopped PD-1s and received hormone therapy. Interestingly, we observed a notable increase in splenic size among some patients compared to baseline during the course of the treatment procedure in the HAIC-LEN-PD1 group. Previous studies have unequivocally illustrated the capacity of oxaliplatin to elicit the onset of sinusoidal obstruction syndrome (SOS), culminating in the manifestation of portal hypertension, fluid retention, and hyperbilirubinemia, ultimately exacerbating the deterioration of hepatic function [33]. Increased splenicsize can be employed as a discerning biomarker, suggesting vulnerability to oxaliplatin-induced hepatic sinusoidal injury. Several patients in the present study experienced hepatic dysfunction despite effective tumor control. Therefore, in addition to assessing the potential hepatotoxicity of lenvatinib and PD-1s, it is crucial to consider the development of SOS and promptly discontinue oxaliplatin administration if necessary. In our department, for patients with refractory thrombocytopenia or spleen size that increased significantly from baseline, partial splentic embolization was performed; however, but the long-term effects should be considered.

Complications associated with portal hypertension, particularly the presence of varices, especially in Vp4 patients, are very important. In this study, by phone follow-up, 18 patients experienced variceal bleeding, 15 of whom were treated with endoscopic hemostasis, 3 of whom were treated with ***percutaneous transsplenic varices embolization (PTSVE)***, and 2 of whom were treated with ***transjugular intrahepatic portosystemic stent shunts (TIPPs)***. In addition, patients with HCC complicated by PVTT often have associated hepatopetal portal venous shunting, exacerbating portal hypertension and potentially leading to liver failure and upper gastrointestinal bleeding. HAIC not only delivers a high local drug concentration into liver tumors directly but can also enter PVTT through hepatopetal portal venous shunting, providing an antitumor effect on the thrombus. In our study, many patients experienced PVTT recanalization, which reduced portal pressure to a certain extent and reduced the risk of bleeding from gastric varices and refractory ascites. Moreover, it is also very important for patient education and medication management. In our center, patients are reminded to pay attention to the color of the stool and go to the hospital in a timely manner once hematemesis and melena occur.

This study had some limitations. First, its retrospective design and non-randomized nature rendered it susceptible to potential biases, despite the absence of disparities in baseline characteristics. Second, the follow-up time was relatively short for OS because an insufficient number of OS events were observed, and long-term survival data are still required. Third, it should be noted that the evaluation of adverse events may not be fully comprehensive due to the retrospective nature of the study, despite our careful inspection of medical records.

To the best of our knowledge, this is the first study to demonstrate improved OS and PFS with HAIC-LEN-PD1 combination treatment versus LEN-PD1 in systemic treatment-naïve high-risk patients with HCC. Moreover, the survival benefit was generally consistent across multiple patient subgroups. Our results revealed that combined HAIC-LEN-PD1 therapy is more effective than LEN-PD1 in controlling intrahepatic tumors and prolonging patient survival. In conclusion, HAIC-LEN-PD1 is a safe and effective treatment for high-risk patients with HCC and provides significant improvements in OS, PFS, and ORR compared to LEN-PD1 with tolerable toxicity.

### Electronic supplementary material

Below is the link to the electronic supplementary material.

Supplementary Material 1

## Data Availability

The dataset used for this study is available from the corresponding author upon reasonable request.

## Abbreviations

HCC: Hepatocellular carcinoma
TACE: Transarterial chemoembolization
PD-1: programmed death receptor-1
PFS: Progression-free survival, OS: Overall survival
PFS: progression-free survival
ORR: Objective response rate
DCR: disease control rate
AFP: Alpha-fetoprotein
HAIC: Hepatic arterial infusion chemotherapy
mRECIST: modified Response Evaluation Criteria in Solid Tumors
CT: computed tomography
CR: complete response
PR: partial response
SD: stable disease
TRAEs: Treatment-related adverse events
TKIs: tyrosine kinase inhibitors
PVTT: portal vein tumor thrombosis
ICIs: Immune checkpoint inhibitors
MDCT: multidetector computed tomography
AEs: adverse events

## References

[1] Sung H, Ferlay J, Siegel RL, Laversanne M, Soerjomataram I, Jemal A, Bray F (2021). Global Cancer statistics 2020: GLOBOCAN estimates of incidence and Mortality Worldwide for 36 cancers in 185 countries. CA Cancer J Clin.

[2] Feng RM, Zong YN, Cao SM, Xu RH (2019). Current cancer situation in China: good or bad news from the 2018 Global Cancer statistics?. Cancer Commun (Lond).

[3] Lin Q, Huang X, Zhong C, Luo T, Zeng X, Chen S (2019). Improved survival with radiotherapy in hepatocellular carcinoma with major vascular invasion: a propensity-matched analysis of Surveillance, Epidemiology, and end results database. Cancer Med.

[4] Zeng H, Chen W, Zheng R, Zhang S, Ji JS, Zou X, Xia C (2018). Changing cancer survival in China during 2003-15: a pooled analysis of 17 population-based cancer registries. Lancet Glob Health.

[5] Siegel RL, Miller KD, Jemal A (2020). Cancer statistics, 2020. CA Cancer J Clin.

[6] Llovet JM, Ricci S, Mazzaferro V, Hilgard P, Gane E, Blanc JF, de Oliveira AC (2008). Sorafenib in advanced hepatocellular carcinoma. N Engl J Med.

[7] Kudo M, Finn RS, Qin S, Han KH, Ikeda K, Piscaglia F, Baron A (2018). Lenvatinib versus Sorafenib in first-line treatment of patients with unresectable hepatocellular carcinoma: a randomised phase 3 non-inferiority trial. Lancet.

[8] Finn RS, Qin S, Ikeda M, Galle PR, Ducreux M, Kim TY, Kudo M (2020). Atezolizumab plus Bevacizumab in Unresectable Hepatocellular Carcinoma. N Engl J Med.

[9] Roy A (2022). Updated efficacy and Safety Data from IMbrave150: Atezolizumab Plus Bevacizumab vs. Sorafenib for Unresectable Hepatocellular Carcinoma. J Clin Exp Hepatol.

[10] Cheng AL, Qin S, Ikeda M, Galle PR, Ducreux M, Kim TY, Lim HY (2022). Updated efficacy and safety data from IMbrave150: Atezolizumab plus Bevacizumab vs. sorafenib for unresectable hepatocellular carcinoma. J Hepatol.

[11] Katagiri S, Yamamoto M (2014). Multidisciplinary treatments for hepatocellular carcinoma with major portal vein tumor thrombus. Surg Today.

[12] Nakazawa T, Hidaka H, Shibuya A, Okuwaki Y, Tanaka Y, Takada J, Minamino T (2014). Overall survival in response to sorafenib versus radiotherapy in unresectable hepatocellular carcinoma with major portal vein tumor thrombosis: propensity score analysis. BMC Gastroenterol.

[13] Finn RS, Ikeda M, Zhu AX, Sung MW, Baron AD, Kudo M, Okusaka T (2020). Phase ib study of Lenvatinib Plus Pembrolizumab in patients with Unresectable Hepatocellular Carcinoma. J Clin Oncol.

[14] Qin S, Chan SL, Gu S, Bai Y, Ren Z, Lin X, Chen Z et al. Camrelizumab plus Rivoceranib versus Sorafenib as first-line therapy for unresectable hepatocellular carcinoma (CARES-310): a randomised, open-label, international phase 3 study. Lancet 2023. 10.1016/S0140-6736(23)00961-3 37499670

[15] Santoni M, Rizzo A, Kucharz J, Mollica V, Rosellini M, Marchetti A, Tassinari E (2023). Complete remissions following immunotherapy or immuno-oncology combinations in cancer patients: the MOUSEION-03 meta-analysis. Cancer Immunol Immunother.

[16] Wang X, Hu J, Cao G, Zhu X, Cui Y, Ji X, Li X (2017). Phase II study of Hepatic Arterial Infusion Chemotherapy with oxaliplatin and 5-Fluorouracil for Advanced Perihilar Cholangiocarcinoma. Radiology.

[17] Hu J, Bao Q, Cao G, Zhu X, Yang R, Ji X, Xu L (2020). Hepatic arterial infusion Chemotherapy using Oxaliplatin plus 5-Fluorouracil Versus Transarterial Chemoembolization/Embolization for the Treatment of Advanced Hepatocellular Carcinoma with Major Portal Vein Tumor thrombosis. Cardiovasc Intervent Radiol.

[18] He M, Li Q, Zou R, Shen J, Fang W, Tan G, Zhou Y (2019). Sorafenib Plus hepatic arterial infusion of Oxaliplatin, Fluorouracil, and Leucovorin vs Sorafenib alone for Hepatocellular Carcinoma with Portal Vein Invasion: a Randomized Clinical Trial. JAMA Oncol.

[19] Boehm LM, Jayakrishnan TT, Miura JT, Zacharias AJ, Johnston FM, Turaga KK, Gamblin TC (2015). Comparative effectiveness of hepatic artery based therapies for unresectable intrahepatic cholangiocarcinoma. J Surg Oncol.

[20] Lyu N, Wang X, Li JB, Lai JF, Chen QF, Li SL, Deng HJ (2022). Arterial chemotherapy of Oxaliplatin Plus Fluorouracil Versus Sorafenib in Advanced Hepatocellular Carcinoma: a biomolecular exploratory, randomized, phase III trial (FOHAIC-1). J Clin Oncol.

[21] Kuo YH, Wu IP, Wang JH, Hung CH, Rau KM, Chen CH, Kee KM (2018). The outcome of sorafenib monotherapy on hepatocellular carcinoma with portal vein tumor thrombosis. Invest New Drugs.

[22] Rizzo A, Ricci AD, Brandi G (2020). Systemic adjuvant treatment in hepatocellular carcinoma: tempted to do something rather than nothing. Future Oncol.

[23] Chuma M, Uojima H, Hiraoka A, Kobayashi S, Toyoda H, Tada T, Hidaka H (2021). Analysis of efficacy of lenvatinib treatment in highly advanced hepatocellular carcinoma with tumor thrombus in the main trunk of the portal vein or tumor with more than 50% liver occupation: a multicenter analysis. Hepatol Res.

[24] Sun X, Zhang Q, Mei J, Yang Z, Chen M, Liang T (2022). Real-world efficiency of lenvatinib plus PD-1 blockades in advanced hepatocellular carcinoma: an exploration for expanded indications. BMC Cancer.

[25] Lai Z, He M, Bu X, Xu Y, Huang Y, Wen D, Li Q (2022). Lenvatinib, toripalimab plus hepatic arterial infusion chemotherapy in patients with high-risk advanced hepatocellular carcinoma: a biomolecular exploratory, phase II trial. Eur J Cancer.

[26] Chang X, Wu H, Ning S, Li X, Xie Y, Shao W, Yu J (2023). Hepatic arterial infusion Chemotherapy Combined with Lenvatinib Plus Humanized programmed death Receptor-1 in patients with high-risk Advanced Hepatocellular Carcinoma: a real-world study. J Hepatocell Carcinoma.

[27] Tanaka K, Yabushita Y, Nakagawa K, Kumamoto T, Matsuo K, Taguri M, Endo I (2013). Debulking surgery followed by intraarterial 5-fluorouracil chemotherapy plus subcutaneous interferon alfa for massive hepatocellular carcinoma with multiple intrahepatic metastases: a pilot study. Eur J Surg Oncol.

[28] de Stefano G, Farella N, Scognamiglio U, Liorre G, Calabria G, Ascione T, Giorgio A (2015). Sorafenib after RFA in HCC patients: a pilot study. Hepatogastroenterology.

[29] Golfieri R, Renzulli M, Mosconi C, Forlani L, Giampalma E, Piscaglia F, Trevisani F (2013). Hepatocellular carcinoma responding to superselective transarterial chemoembolization: an issue of nodule dimension?. J Vasc Interv Radiol.

[30] Khalaf MH, Sundaram V, AbdelRazek Mohammed MA, Shah R, Khosla A, Jackson K, Desai M (2019). A predictive model for postembolization syndrome after transarterial hepatic chemoembolization of Hepatocellular Carcinoma. Radiology.

[31] Lopez-Benitez R, Richter GM, Kauczor HU, Stampfl S, Kladeck J, Radeleff BA, Neukamm M (2009). Analysis of nontarget embolization mechanisms during embolization and chemoembolization procedures. Cardiovasc Intervent Radiol.

[32] Fu Y, Peng W, Zhang W, Yang Z, Hu Z, Pang Y, Hu D (2023). Induction therapy with hepatic arterial infusion chemotherapy enhances the efficacy of lenvatinib and pd1 inhibitors in treating hepatocellular carcinoma patients with portal vein tumor thrombosis. J Gastroenterol.

[33] Overman MJ, Maru DM, Charnsangavej C, Loyer EM, Wang H, Pathak P, Eng C (2010). Oxaliplatin-mediated increase in spleen size as a biomarker for the development of hepatic sinusoidal injury. J Clin Oncol.

