# Supplementary material for: HAIC Combined with lenvatinib plus PD-1 versus lenvatinib Plus PD-1 in patients with high-risk advanced HCC: a real-world study
Source: BMC Cancer. 2024 Apr 16;24:480. doi: 10.1186/s12885-024-12233-6 (PMC11020267; doi:10.1186/s12885-024-12233-6)
Supplement: Supplementary file 1 — Supplementary Material 1 [file 12885_2024_12233_MOESM1_ESM.docx]

**Supplementary material 1a**

**

**

Flow chart of the disposition process of HCC patients.

**Supplementary material 1b**

Kaplan-Meier analyses of progression-free survival (PFS) and overall survival (OS) according to different PD-1 inhibitors in the patients receiving HAIC-LEN-PD1 and LEN-PD1.The median PFS (**a**) of the camrelizumba, tisleizumab and sintilimab was 9.1 months (95%CI 7.5–10.7) , 9.9 months (95%CI 7.9–11.8), and 7.7 months (95%CI 3.4–12.1) in HAIC-LEN-PD1, respectively (*p*=0.802).The median PFS (**b**) of the camrelizumba, tisleizumab and sintilimab was 4.5 months (95%CI 3.2–5.8), and 4.8 months (95%CI 3.8–5.8) , 6.9 months (95%CI 3.4–10.3) in LEN-PD1, respectively (*p*=0.544).The median OS (**c**) of the camrelizumba, tisleizumab and sintilimab was 21.7 months (95%CI 12.0–31.3) , 15.3 months (95%CI 11.7–18.8), and NE in HAIC-LEN-PD1, respectively (*p*=0.641).The median OS (**d**) of the camrelizumba, tisleizumab and sintilimab was 7.7 months (95%CI 5.25–10.28) , 10.1 months (95%CI 4.5–15.8), and 18.4 months (95%CI 0.1–36.6) in LEN-PD1, respectively (*p*=0.292). NE; not estimable.

| Univariable and multivariable analyses of covariables associated with PFS and OS | | | | | | | | | | | | | | | | |
| --- | --- | --- | --- | --- | --- | --- | --- | --- | --- | --- | --- | --- | --- | --- | --- | --- |
| Variable |  | PFS | | | | | | |  | OS | | | | | | |
|  |  | Univariable analysis | | |  | Multivariable analysis | | |  | Univariable analysis | | |  | Multivariable analysis | | |
|  |  | HR | 95%CI | *P*-value |  | HR | 95%CI | *P*-value |  | HR | 95%CI | *P*-value |  | HR | 95%CI | *P*-value |
| Gender  (Female vs Male) |  | 0.728 | 0.418-1.269 | 0.263 |  |  |  |  |  | 0.432 | 0.233-0.799 | **0.007** |  | 0.255 | 0.132-0.491 | **＜0.001** |
| Age  (<60 vs ≥60) |  | 0.751 | 0.517-1.089 | 0.131 |  |  |  |  |  | 0.784 | 0.481-1.277 | 0.328 |  |  |  |  |
| AFP  (≤400 vs >400 ng/ml) |  | 1.059 | 0.741-1.513 | 0.753 |  |  |  |  |  | 1.246 | 0.789-1.966 | 0.345 |  |  |  |  |
| Child-Pugh class  (A vs B) |  | 1.456 | 0.957-2.215 | 0.079 |  | 1.151 | 0.732-1.809 | 0.543 |  | 2.294 | 1.415-3.720 | **0.001** |  | 2.179 | 1.156-4.106 | **0.016** |
| ALBI  （Grade1 vs Grade2/3） |  | 1.514 | 1.071-2.139 | **0.019** |  | 1.262 | 0.866-1.838 | 0.225 |  | 2.183 | 1.378-3.458 | **0.001** |  | 1.615 | 0.971-2.684 | 0.065 |
| HBV infected  ( No vs Yes ) |  | 1.085 | 0.621-1.893 | 0.775 |  |  |  |  |  | 1.133 | 0.546-2.353 | 0.737 |  |  |  |  |
| Extrahepatic metastasis  (No vs Yes) |  | 1.765 | 1.234-2.526 | **0.002** |  | 1.497 | 1.035-2.165 | **0.032** |  | 1.894 | 1.223-2.935 | **0.004** |  | 1.510 | 0.962-2.371 | 0.073 |
| Vp4 (No vs Yes) |  | 0.699 | 0.496-0.986 | **0.041** |  | 0.775 | 0.546-1.101 | 0.155 |  | 0.715 | 0.463-1.104 | 0.130 |  |  |  |  |
| TO≥50% (No vs Yes) |  | 1.235 | 0.848-1.799 | 0.272 |  |  |  |  |  | 0.945 | 0.577-1.548 | 0.822 |  |  |  |  |
| Vp4 and TO≥50%  (No vs Yes) |  | 1.392 | 0.900-2.152 | 0.138 |  |  |  |  |  | 1.781 | 1.085-2.924 | **0.022** |  | 1.068 | 0.585-1.949 | 0.830 |
| Combined HAIC  (No vs Yes) |  | 0.483 | 0.341-0.685 | **＜0.001** |  | 0.516 | 0.362-0.736 | **＜0.001** |  | 0.429 | 0.278-0.662 | **＜0.001** |  | 0.350 | 0.218-0.562 | **＜0.001** |

**Supplementary material 1c**

**Supplementary material 1d**

| **Further treatments after disease progression** | | | |  |
| --- | --- | --- | --- | --- |
|  | HAIC-LEN-PD1 (n=103) | LEN-PD1 (n=61) | *P*-value | |
| Disease Progression | 78 | 55 |  | |
| Best Supportive Care | 17 | 8 | 0.559 | |
| HAIC+ regorafenib | 4 | 6 | 0.282 | |
| HAIC+regorafenib+PD-1 | 10 | 7 | 0.803 | |
| TACE+regorafenib | 7 | 5 | 0.867 | |
| TACE+regorafenib+PD-1 | 16 | 9 | 0.362 | |
| Atezolizumabplusbevacizumab | 3 | 2 | 0.856 | |
| Regorafenib | 2 | 2 | 0.803 | |
| Regorafenib+PD1 | 6 | 10 | 0.105 | |
| SBRT+regorafenib and/or PD-1 | 6 | 3 | 0.503 | |
| others | 7 | 3 | 0.351 | |
| HAIC:hepatic arterial infusion chemotherapy;TACE:Transarterial chemoembolization; PD-1:humanized programmed death receptor-1; SBRT: stereotactic body radiationtherapy | | | |  |

**Supplementary material 1e**


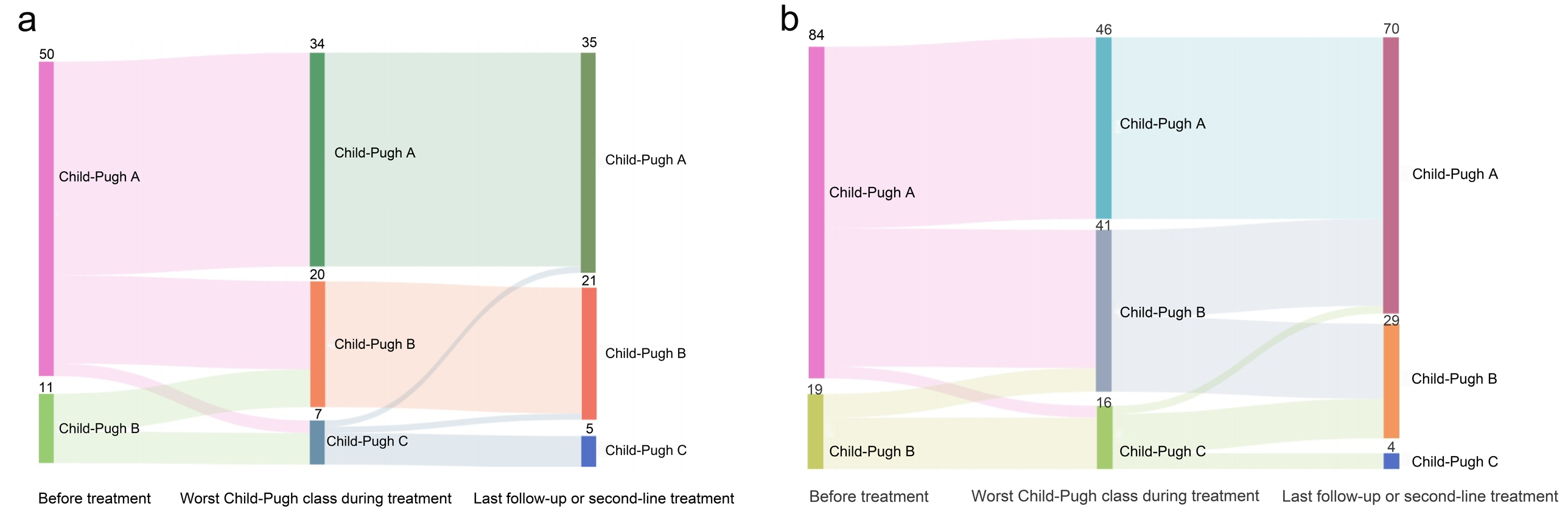


The Sankey diagram of the dynamic changes of Child-Pugh class in the patients receiving LEN-PD1 **(a)** and HAIC-LEN-PD1 **(b)**. The number above each bar represents the corresponding number of patients.

**Supplementary material 1f**

| Treatment related adverse events of different immune checkpoint inhibitors | | | | | | | |
| --- | --- | --- | --- | --- | --- | --- | --- |
| **Adverse event** | **Any Grade** | | |  | **Grade 3-4** | | |
|  | Tislelizumab | Sintilimab | Camrelizumab |  | Tislelizumab | Sintilimab | Camrelizumab |
| Hypothyroidism | 16 | 8 | 34 |  | 2 | 1 | 2 |
| Immune-related hepatitis | 2 | 1 | 4 |  | 1 | 0 | 2 |
| Immune-related pneumonitis | 1 | 1 | 3 |  | 1 | 0 | 1 |
| Immune-related dermatitis | 2 | 2 | 6 |  | 1 | 0 | 1 |
| Immune-related myocarditis | 0 | 1 | 0 |  | 0 | 1 | 0 |
